# Risk of recurrence, subsequent mode of birth and morbidity for women who experienced severe perineal trauma in a first birth in New South Wales between 2000 –2008: a population based data linkage study

**DOI:** 10.1186/1471-2393-13-89

**Published:** 2013-04-08

**Authors:** Holly Priddis, Hannah G Dahlen, Virginia Schmied, Annie Sneddon, Christine Kettle, Chris Brown, Charlene Thornton

**Affiliations:** 1 School of Nursing and Midwifery, University of Western Sydney, Locked Bag 1797, Penrith South DC, NSW, Australia; 2 The Gold Coast Health Services District, Griffith University, 108 Nerang Street, Southport, QLD, 4215, Australia; 3 Faculty of Health, Staffordshire University, Blackheath Lane, Beaconside, Stafford, ST18 0AD, UK; 4 NHMRC Clinical Trials Centre, Locked Bag 77, Camperdown, NSW, 1450, Australia

**Keywords:** Severe perineal trauma, Subsequent birth, Postpartum morbidities, Risk of recurrence

## Abstract

**Background:**

Severe perineal trauma occurs in 0.5-10% of vaginal births and can result in significant morbidity including pain, dyspareunia and faecal incontinence. The aim of this study is to determine the risk of recurrence, subsequent mode of birth and morbidity for women who experienced severe perineal trauma during their first birth in New South Wales (NSW) between 2000 – 2008.

**Method:**

All singleton births recorded in the NSW Midwives Data Collection between 2000–2008 (n=510,006) linked to Admitted Patient Data were analysed. Determination of morbidity was based upon readmission to hospital within a 12 month time period following birth for a surgical procedure falling within four categories: 1. Vaginal repair, 2. Fistula repair, 3. Faecal and urinary incontinence repair, and 4. Rectal/anal repair. Women who experienced severe perineal trauma during their first birth were compared to women who did not.

**Results:**

2,784 (1.6%) primiparous women experienced severe perineal trauma during this period. Primiparous women experiencing severe perineal trauma were less likely to have a subsequent birth (56% vs 53%) compared to those not who did not (OR 0.9; CI 0.81-0.99), however there was no difference in the subsequent rate of elective caesarean section (OR 1.2; 0.95-1.54), vaginal birth (including instrumental birth) (OR 1.0; CI 0.81-1.17) or normal vaginal birth (excluding instrumental birth) (OR 1.0; CI 0.85-1.17). Women were no more likely to have a severe perineal tear in the second birth if they experienced this in the first (OR 0.9; CI 0.67-1.34). Women who had a severe perineal tear in their first birth were significantly more likely to have an ‘associated surgical procedure’ within the ≤12 months following birth (vaginal repair following primary repair, rectal/anal repair following primary repair, fistula repair and urinary/faecal incontinence repair) (OR 7.6; CI 6.21-9.22). Women who gave birth in a private hospital compared to a public hospital were more likely to have an ‘associated surgical procedure’ in the 12 months following the birth (OR 1.8; CI 1.54-1.97), regardless of parity, birth type and perineal status.

**Conclusion:**

Primiparous women who experience severe perineal trauma are less likely to have a subsequent baby, more likely to have a related surgical procedure in the 12 months following the birth and no more likely to have an operative birth or another severe perineal tear in a subsequent birth. Women giving birth in a private hospital are more likely to have an associated surgical procedure in the 12 months following birth.

## Background

Severe trauma to the perineum during vaginal birth can occur spontaneously or as a result of obstetric intervention [1,2]. Severe perineal trauma (SPT) is defined as a third degree tear which involves injury to the perineum involving the anal sphincter complex (this is further graded as 3a, 3b or 3c depending on the extent of external and internal anal sphincter involvement); or a fourth degree tear which involves injury to the perineum involving the external, internal and epithelium of the anal sphincter [3].

While approximately two thirds of Australian women will experience some trauma to the perineum during a vaginal birth [4], around 1.7% experience severe perineal trauma (SPT). This rate ranges from 1.5% in Queensland, to 2.3% in the Australian Capital Territory [4]. While exact figures reporting the incidence of severe perineal trauma, with associated damage to the anal sphincter, are unclear, on an international scale the incidence is reported to range from between 0.5 – 10% [5,6]. Andrews et al. (2006) report that the incidence of severe perineal trauma increases to up to 19% in centres where midline episiotomies are performed [7].

### Risk factors for severe perineal trauma

Risk factors associated with an increased incidence of severe perineal trauma include parity (primiparous), maternal age (very young and old), nutritional status, previous experience of perineal trauma, fetal weight, abnormal collagen synthesis and gender of the fetus [5,8,9]. Whilst literature reports that women of Asian ethnicity are at an increased risk of sustaining severe perineal trauma when giving birth in Western countries, controversy remains as to whether this risk remains when these women birth within their country of origin [10,11]. Intrapartum risk factors include fetal presentation, episiotomy (particularly midline), instrumental birth, prolonged second stage of labour, birth position during second stage, and obstetric emergencies such as shoulder dystocia [8,12-15].

It has been suggested that women who have experienced severe perineal trauma may be fearful of experiencing a subsequent pregnancy and birth; this fear is reported to be based upon the risk of sustaining subsequent perineal trauma, and that these women may require an episiotomy or a caesarean section [16,17]. Some women who experience severe perineal trauma express a preference for a vaginal birth subsequently, despite their fears regarding risk of recurrence [16]. There are contradictory findings reported in the literature regarding the risk of recurrence for women who have experienced third or fourth degree perineal trauma [18-21]. A review conducted by Edwards et al. (2006) reviewed 271 cases, from 1991 to 2003, of women who experienced a subsequent labour and birth following severe perineal trauma with the first birth. The authors reported that the rate of recurrence was not statistically significant when compared to women experiencing initial severe perineal trauma (2.4% vs 3.3%, OR 0.72, 95% CI 0.33 – 1.59). Of those that did go on to experience recurrent trauma, risk factors included age, weight of the woman, birth weight of the newborn, the use of episiotomy and instrumental births [19]. A cohort study conducted by Baghestan et al. (2011) reported that an obstetric history of severe perineal trauma in the first or second birth increased the likelihood of repeat trauma occurring in the third birth. Likewise women who experienced severe perineal trauma were less likely to have a second pregnancy compared with women who had no history of severe perineal trauma (66.7% versus 76.9%); however these findings were not tatistically significant [18]. The risk is reported to increase in large maternity units (>3000 births per annum), when the newborn weight is above 3500 grams, and when forceps are used during the second stage of labour [18,19,21].

The aim of this study was to determine the risk of recurrence, subsequent mode of birth and morbidity for women who experienced severe perineal trauma in a first birth in NSW between 2000–2008.

## Methods

### Data sources

Birth data for the time period July 1st 2000 until June 30th 2008 of all singleton births was provided by NSW Department of Health as recorded in the NSW Midwives Data Collection (MDC). This legislated, population based surveillance system contains maternal and infant data on all births of ≥400 grams birth weight or ≥20 weeks gestation.

The recording of perineal status was altered on the MDC in 2006. Prior to 2006, perineal status was recorded as intact/graze, 1st degree tear, 2nd degree tear, 3rd degree, 4th degree tear, episiotomy and combined episiotomy and tear. Post 2006 combined episiotomy and tear was removed. The two versions of the data were merged for the purpose herein. The data item ‘Episiotomy Yes/No’ was also utilised. The accuracy of the recording of perineal status has previously been shown to have a kappa of 0.84 and 0.82 in two separate and individual studies [22,23]. The positive predictive value (PPV) of 1st, 2nd, 3rd and 4th degree tears have been reported as 76.6, 96.6, 72.8 and 100.0 respectively. This PPV provides an overview of the validity of the recording of perineal status in various sources including electronic and paper based medical records. Only women recorded as having a vaginal birth were included in this study.

Data on all hospital admissions was provided by the census data collection, the Admitted Patient Data Collection (APDC). The clinical data component of the APDC utilises the International Classification of Diseases – Australian modification (ICD-10-AM). Probabilistic linkage of the two datasets was undertaken by the Centre for Health Record Linkage. The validity and accuracy of this process has been examined and these datasets have low rates of missing data when compared to medical records and high levels of agreement [22,23].

Seven thousand APDC codes related to all patient admissions over the period from 2000–2008 in NSW were sorted individually by the first author, and coded under sixteen sub headings. The purpose of sorting these codes was to determine the reason for, and frequency of, admission for women within a 12 month time period following birth for procedures related to perineal/pelvic floor trauma. The identified subheadings were: Nervous System; Skin; Skeleton; Renal/Ureter/Bladder; Fertility/Pregnancy; Miscellaneous (this included such codes as oncology related therapies); Eyes; Ears; Respiratory; Cardiac; Gastrointestinal; Pelvic/Sphincter/Urethral; Lymphatics; Breast; Psychiatric and Male Specific Codes. Any procedural codes relating to diagnosis and repair of the initial trauma were not included in the final coding categories. A list of potential Medicare Benefit Schedule (MBS) procedural codes was identified by the first author. This initial sorting of codes was completed and independently reviewed by two of the co-authors for accuracy. This list was then reviewed independently by three specialists in fields related to perineal trauma and outcomes including: 1. A midwife running a postnatal specialist perineal trauma clinic, 2. An obstetrician, and 3. A colorectal surgeon. Through this consensus process a refined list of codes that were specifically associated with therapies and treatment for morbidities potentially occurring as a result of severe perineal trauma from subheadings Renal/Ureter/Bladder, Fertility/Pregnancy, and Pelvic/Sphincter/Urethral was agreed upon. These codes were then grouped into the following four categories related by procedure and physiology: 1. Vaginal repair, 2. Fistula repair, 3. Faecal and urinary incontinence repair, and 4. Rectal/anal repair. There were 34 subgroups/diagnostic codes for vaginal repair, eight for fistula, eight for faecal and urinary incontinence and 11 for rectal/anal repair.

The first pregnancy recorded in the MDC with vaginal birth documented as the mode of birth was considered the index pregnancy for this dataset regardless of parity. For this reason, sub-analyses were undertaken according to parity.

Ethical approval was obtained from the NSW Population and Health Services Research Ethics Committee, Protocol No.2010/12/291.

### Data analysis

Descriptive analyses of short and long term morbidity associated with all types of perineal trauma was produced utilising SPSS v.19 (IBM). Frequency distributions were used to classify the population and descriptive statistics the morbidity outcomes. Relative risk was calculated between factors and events, Odds Ratios (OR) are reported for rare outcomes. Due to the number of associations examined, the level of statistical significance was set at <0.001.

## Results

Between July 1st 2000 and June 30th 2008 there were 510,006 vaginal births. Nearly all of these births occurred in hospital (95%) and 71% of the women were born in Australia. Of the women giving birth vaginally 14.2% had an instrumental birth and 0.6% had a vaginal breech birth (Table 1).

**Table 1:** Demographics and mode of birth of women giving birth in NSW between 2000-2008

	**All women**
No of births	510,006
Age of women at delivery (Mean and SD)	29.7 (5.55)
% Primiparous	40.6%
Place of birth	
Hospital	94.5%
Birth Centre	3.30%
Planned Birth Centre transferred to hospital	1.30%
Planned Home Birth	0.20%
Planned Home Birth transferred to Hospital	0.01%
Born Before Arrival	0.60%
Type of Birth	
Normal Vaginal Delivery	85.3%
Forceps	4.8%
Ventouse	9.4%
Vaginal Breech	0.6%
Country of Birth of Mother	
Australia	71.6%
New Zealand	2.6%
England	2.2%
Vietnam	2.2%
China	2.1%
Lebanon	2.1%
Other	17.2%

**Figure 1:**
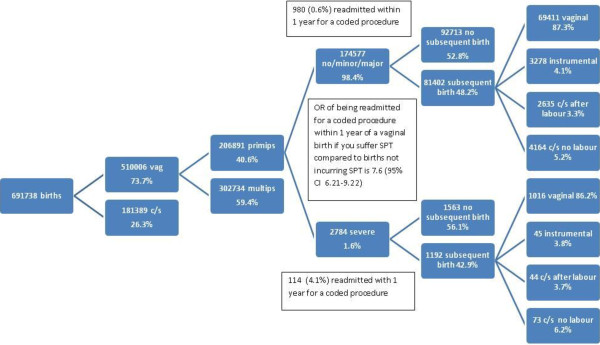
Flow diagram of mode of birth and perineal outcomes following index birth with severe perineal trauma and without.

The overall incidence of severe perineal trauma in women giving birth to singleton infants from 2000–2008 was 1.6% (n=2784) for primiparous women (Figure 1). Primiparous women experiencing severe perineal trauma were less likely to have a subsequent birth (56% vs 53%) compared to those not experiencing it (OR 0.9; CI 0.81-0.99). There was no difference in the rate of elective caesarean section (OR 1.2; 0.95-1.54), vaginal birth (including instrumental birth) (OR 1.0; CI 0.81-1.17) or normal vaginal birth (excluding instrumental birth (OR 1.0; CI 0.85-1.17). Women were no more likely to have a severe perineal tear in the second birth if they experienced this in the first (OR 0.9; CI 0.67-1.34) (Table 2).

**Table 2:** Outcomes for women in subsequent births following a previous severe perineal trauma

	**OR**	**p**
Any subsequent birth	**0.9 (0.81-0.99)**	**<0.001**
Elective C/S	1.2 (0.95-1.54)	0.95
Vaginal birth rate	1.0 (0.81-1.17)	0.39
Normal vaginal birth outcome	1.0 (0.85-1.17)	0.50
SPT in second delivery	0.9 (0.67-1.34)	0.74

Primiparous women who had a severe perineal tear in their first birth were significantly more likely to have an ‘associated surgical procedure’ in the 12 months following birth (vaginal repair following primary repair, rectal/anal repair following primary repair, fistula repair and urinary/faecal incontinence repair) compared to women who did not have a severe perineal tear (4.1% vs 0.6%). In the second pregnancy following the index birth where the primary severe perineal trauma occurred, 12 women following a vaginal birth (1.1%) and one woman following a caesarean section (0.85%) had an admission ≤12 months following the second birth for an ‘associated surgical procedure’.

Overall, both primiparous and multiparous women admitted for an ‘associated surgical procedure’ ≤12 months post birth were more likely to be older (29.7 vs 30.8), primiparous (49.5% vs 40.6%), not to smoke (11.9% vs 15.8%) and have a private hospital admission for the procedure (34.5% vs 20.7%) (Table 3). Women who gave birth in a private hospital during the eight year period were more likely to have an ‘associated surgical procedure’ in the 12 months following the birth (OR 1.8; CI 1.54-1.97), regardless of parity, birth type and perineal status.

**Table 3:** Demographic and medical factors associated with admission for an associated surgical procedure in the 12 months following a birth

	**All vaginal births**	**Admitted within 12 months post birth**	**p**
N	507508	2498	
Age (years)	29.7 (5.55SD) (12–55 range)	30.8 (5.14SD) (15–48 range)	<0.001
% Primiparous	40.6%	49.5%	<0.001
Smoking	15.8%	11.9%	<0.001
Hypertensive Disorder of Pregnancy	4.8%	4.4%	NS
Gestational diabetes	3.9%	3.6%	NS
Private hospital admission	20.7%	34.5%	<0.001

## Discussion

The data in this study indicates that women who experience severe perineal trauma with their first birth were significantly less likely to experience a subsequent pregnancy and birth during the 8 year period (56% versus 53%). Similar findings have been reported by Baghestan et al. (2011) and Elfaghi et al. (2004). Elfaghi et al. (2004) reported this finding as concerning, and therefore suggested that providing women with the option of elective caesarean sections may result in increased subsequent pregnancies for women who had experienced previous birth trauma [21]. Whilst the findings reported above are statistically significant, the reported difference between groups may have limited clinical significance. Further, it is possible that the women who gave birth in 2008 in the dataset have experienced a subsequent pregnancy and birth after data collection ceased. Removal of these women from the dataset was considered however due to the relatively rare occurrence of severe perineal trauma it was decided to include these women.

A prospective cohort study conducted by Gottvall and Waldenstrom (2002) investigated whether the experience of the first birth impacted upon women choosing to experience subsequent pregnancies and births [24]. The authors reported that women who described their first birth experience as negative were significantly less likely to experience a subsequent pregnancy, and for those that did, there was a larger interval between the first and second births which supports the findings of this study [24]. In a qualitative study conducted by Rilby et al. (2012) exploring the feelings and fears experienced by women planning a subsequent pregnancy and birth, while women report feeling fearful particularly in relation to anticipated pain or potential complications to either themselves or the newborn baby, they were motivated by the positive experience of a vaginal birth and the newborn baby. Some women however did report that due to their fears and previous experiences, they would opt for a caesarean birth [25].

### Elective caesarean or normal vaginal birth in subsequent pregnancy

The findings of this study report that for women who experience third and/or fourth degree perineal trauma there is no difference between elective caesarean section and normal vaginal birth rate for subsequent deliveries. For women who are symptomatic for urinary, flatus or faecal incontinence, or have findings that deviate from normal via manometric or endoanal ultrasonography associated with severe perineal trauma, it is suggested that an elective caesarean section should be recommended by the health care professional [3]. However, debate continues as to the most appropriate management of the subsequent mode of birth following severe perineal trauma, with discussions focussed around the protective mechanisms, potential morbidities and increased risk of subsequent perineal trauma associated with operative vaginal and vaginal births [3,18,20,21,26].

Concerns exist as to the ongoing integrity of the pelvic floor and anal sphincter function if a woman is to experience a subsequent vaginal birth [27]. In a study conducted by McKenna et al. (2003) looking at outcomes related to elective caesarean sections for women who have a history of severe perineal trauma, the authors suggested that the risks associated with a repeat outcome of severe perineal trauma and the associated potential morbidities such as faecal incontinence outweighs the potential morbidities that can occur as a result of an elective caesarean section [28]. In contrast, a study conducted by Scheer et al. (2009) investigated sphincter integrity via manometric assessment, associated function, and quality of life for women who were asymptomatic (n= 73), who experienced a subsequent birth following severe perineal trauma. The authors reported that there were no significant differences in sphincter integrity via manometry, or quality of life as reported by survey, following a subsequent vaginal birth; three women experienced repeat severe perineal trauma and one new internal sphincter defect was detected [26]. The data in our study was limited by small numbers so we could not examine morbidity in future pregnancies. In the second pregnancy following the index birth where the primary severe perineal trauma occurred, only 12 women following a vaginal birth (1.1%) and one woman following a caesarean section (0.85%) had an admission ≤12 months following the second birth for an ‘associated surgical procedure’.

There is also some evidence to suggest that following traumatic birth experiences, women may choose a vaginal birth over a caesarean section for subsequent births, and it is suggested that these choices may occur as a result of an understanding of the physiological and psychological benefits for both themselves and their newborns [16,29,30]. Women further report the benefits of vaginal birth as enabling initial bonding with their newborn infant; this is seen as particularly important by women who are planning a vaginal birth after caesarean [17,31]. It has been reported that women identify a strong link between labour and vaginal birth, femininity, and as a rite of passage to womanhood [30,31].

### Risk of repeat SPT

In this study, in comparison to women who had not sustained severe perineal trauma during birth, women who experienced a third of fourth degree perineal tear were no more likely to experience severe perineal trauma in a subsequent pregnancy. Studies investigating the risk of recurrence of in subsequent births for women who have experienced severe perineal trauma vary widely in findings [18-20]. Studies by Edwards et al. (2006) and Scheer et al. (2009) support the findings in this study, while Lowder (2007) reported that women who had experienced severe perineal trauma with their index pregnancy, were at increased risk of subsequent perineal trauma in comparison to women with no history of perineal trauma (7.2% versus 2.3%). This risk of recurrence was increased as a result of episiotomy, malpresentation, shoulder dystocia, and birth weight greater than 3500 grams [19,20,26]. These associations have also been reported elsewhere [18,20,32].

### Sociodemographic influences

In a previous study we found that women who gave birth in a private hospital compared to a public hospital were at greater risk of SPT compared to women who had no perineal trauma or minor perineal trauma (1st degree, vaginal/labial tear); however they were at no increased risk when compared to all women who did not experience SPT (9). Other studies have shown a protective effect of private hospital care on SPT rates [33] but have been criticised for methodological flaws, such as not including extensions of episiotomy in the data examined [34]. There are also concerns expressed by health practitioners that SPT is under-reported in private hospitals [35]. In this study we found that women who gave birth in a private hospital regardless of parity, mode of birth or degree of perineal trauma were more likely to be admitted for an ‘associated surgical procedure’ in the ≤ 12 months following birth. This could indicate under-reporting of severe perineal trauma in private hospitals or alternatively a higher tendency for those women who are socially advantaged to access surgical procedures following birth. We also noted older age, primiparity and non-smoking increased the chance of admission for an ‘associated surgical procedure’. While age and parity have been associated with increased risk of SPT in the literature [36] smoking has not, though it has been associated with lower birth weight babies [37]. Again these could be more social markers than causal factors with socially advantaged women being more informed about health care options and being more likely to be older and non-smoking.

### Best practice

It has been suggested that best practice models of care for women who sustain severe perineal trauma include a referral for an endoanal ultrasound and consultation with a colorectal surgeon in locations where specialist perineal care clinics are operational, and women can access supportive, collaborative care within the one facility [38].

### Limitations

There are significant advantages of using population based datasets such as the MDC, including the size of the dataset, a well validated dataset and the anonymous nature of the results therein. The limitations are the limited number of variables that are included and the scarcity of specific information on potential confounders, specifically information regarding maternal weight and pregnancy weight gain and pre-existing medical conditions. This paper has reported that older age is associated with admission for an associated surgical procedure (30 years versus 31 years); whilst these findings are statistically significant the clinical significance is questionable. We are reassured by previous validation studies that perineal status is very accurately recorded [23,39,40]. There is also under reporting in some organisations making accurate ascertainment and comparison of SPT rates difficult.

## Conclusion

In this study we found primiparous women experiencing severe perineal trauma compared to those who did not were less likely to have a subsequent birth, no less likely to have a normal vaginal birth in a subsequent pregnancy and no more likely to have a repeat SPT. Women who had a severe perineal tear in their first birth were however significantly more likely to have an ‘associated surgical procedure’ in the ≤12 months following birth and women who gave birth in a private hospital are also more likely to have an ‘associated surgical procedure’ in the 12 months following the birth. More research is needed to explore with women and health providers their decision making following a SPT to determine whether women are making the choice of a vaginal birth following a SPT or if health practitioners are influencing this choice. There is also an urgent need to explore the experiences of women who experience a SPT and the impact of the morbidity observed in this study on their wellbeing. There is also limited information available about the ideal construction of health services for women experiencing SPT from the perspective of women and health providers.

## Competing interests

The authors declare that they have no competing interests.

## Authors’ contributions

HP participated in data review, design and drafting of manuscript as a component of a doctoral study. HD conceived of the study design, participated in design and drafting of manuscript. VS assisted with review of data. CT conducted statistical analysis and helped review the manuscript draft. CB advised and assisted with statistical analysis. AS, CK and MG provided expert review of coded data and assisted with writing the manuscript. All authors read and approved the final manuscript.

## Pre-publication history

The pre-publication history for this paper can be accessed here:

http://www.biomedcentral.com/1471-2393/13/89/prepub
